# Mobilisation and remobilisation of a large archetypal pathogenicity island of uropathogenic *Escherichia coli in vitro *support the role of conjugation for horizontal transfer of genomic islands

**DOI:** 10.1186/1471-2180-11-210

**Published:** 2011-09-24

**Authors:** György Schneider, Ulrich Dobrindt, Barbara Middendorf, Bianca Hochhut, Valéria Szijártó, Levente Emődy, Jörg Hacker

**Affiliations:** 1 Institute for Molecular Infection Biology, University of Würzburg, Josef-Schneider-Str. 2/Building D15, Würzburg, 97070, Germany; 2 Institute of Medical Microbiology and Immunology, University of Pécs, Szigeti ut 12, Pécs, 7624, Hungary; 3 Veterinary Medical Research Institute, Hungarian Academy of Sciences, Hungária krt. 21 Budapest, 1143, Hungary; 4 Institute for Hygiene, University of Münster, Josef-Schneider-Str. 41, Münster, 48149, Germany; 5 German Academy of Sciences Leopoldina, Emil-Abderhalden-Str. 37, Halle (Saale), 06019, Germany

## Abstract

**Background:**

A substantial amount of data has been accumulated supporting the important role of genomic islands (GEIs) - including pathogenicity islands (PAIs) - in bacterial genome plasticity and the evolution of bacterial pathogens. Their instability and the high level sequence similarity of different (partial) islands suggest an exchange of PAIs between strains of the same or even different bacterial species by horizontal gene transfer (HGT). Transfer events of archetypal large genomic islands of enterobacteria which often lack genes required for mobilisation or transfer have been rarely investigated so far.

**Results:**

To study mobilisation of such large genomic regions in prototypic uropathogenic *E. coli *(UPEC) strain 536, PAI II_536 _was supplemented with the *mob*_RP4 _region, an origin of replication (*oriV*_*R6K*_), an origin of transfer (*oriT*_*RP4*_) and a chloramphenicol resistance selection marker. In the presence of helper plasmid RP4, conjugative transfer of the 107-kb PAI II_536 _construct occured from strain 536 into an *E. coli *K-12 recipient. In transconjugants, PAI II_536 _existed either as a cytoplasmic circular intermediate (CI) or integrated site-specifically into the recipient's chromosome at the *leuX *tRNA gene. This locus is the chromosomal integration site of PAI II_536 _in UPEC strain 536. From the *E. coli *K-12 recipient, the chromosomal PAI II_536 _construct as well as the CIs could be successfully remobilised and inserted into *leuX *in a PAI II_536 _deletion mutant of *E. coli *536.

**Conclusions:**

Our results corroborate that mobilisation and conjugal transfer may contribute to evolution of bacterial pathogens through horizontal transfer of large chromosomal regions such as PAIs. Stabilisation of these mobile genetic elements in the bacterial chromosome result from selective loss of mobilisation and transfer functions of genomic islands.

## Background

In the early 1980s large unstable chromosomal regions carrying virulence-associated genes were identified in uropathogenic *E. coli *[1]. Later, such large unstable chromosomal regions were designated pathogenicity islands (PAIs) [2-4]. A constantly increasing number of similar genetic elements detected in many pathogenic and non-pathogenic microorganisms led to the definition of a family of related genetic elements, termed genomic islands (GEIs), whose members share characteristic features [5-7]. Although PAIs, a subgroup of GEIs, are in several cases superficially similar, they structurally differ with respect to the encoded virulence factors, the size and the presence of different mobile and accessory elements. Due to the presence of mobility genes (integrases, transposases, IS elements) or the occurrence of recombination processes or point mutations, PAIs constantly undergo structural changes [4,8-12]. Upon acquisition and chromosomal insertion, islands together with additional large regions of flanking chromosomal sequence context can be transferred by conjugation and homologous recombination and thus contribute to genome plasticity and the simultaneous transfer of multiple traits [13].

Nevertheless, PAIs are in many cases not stably integrated into the *E. coli *host chromosome and may be lost upon deletion. This process can be studied by island probing [10,14-16]. The influence of different environmental conditions on the stability of five PAIs of UPEC strain 536 has already been investigated before [17] indicating that PAI I_536_, PAI II_536_, PAI III_536_, and PAI V_536 _delete with frequencies between 10^-5 ^and 10^-6^, while loss of PAI IV_536 _could not be detected. In UPEC strain 536, PAI deletion is catalyzed by a P4-like bacteriophage integrase which is encoded on the respective island [18]. Similar deletion frequencies (10^-5 ^- 10^-6^) were also reported for PAIs of REPEC strain 84/110-1 and *S. flexneri *2a [12,19]. Higher deletion frequencies (10^-3 ^- 10^-4^) have, however, been observed for O-islands 43 and 48 in enterohemorrhagic *E. coli *(EHEC) O157:H7 isolates [14]. Circular intermediate (CI) formation in the cytoplasm of UPEC strain 536 was demonstrated for PAI II_536 _and PAI III_536_. Since none of these two islands apparently contain an origin of replication, it has been hypothesized that CIs are lost upon cell division unless they reintegrate into the chromosome. Furthermore, horizontal gene transfer (HGT) of such circularized PAIs may occur with the help of bacteriophages or conjugative plasmids [17].

A close functional association between PAIs and bacteriophages was reported for several bacterial pathogens. In *V. cholerae*, the entire 39.5-kb *Vibrio *Pathogenicity Island (VPI) can be transfered by the general transducing phage CP-T1 [20]. The "high pathogenicity island (HPI)" of *Yersinia pseudotuberculosis *has been shown to be transfered by a bacteriophage [21]. The so-called *Staphylococcus aureus *pathogenicity islands (SaPIs) can excise and replicate upon induction by other resident *S. aureus *bacteriophages and then packed into phage-like particles which can be transferred to recipient cells [22-24]. SaPI transfer by transduction can even occur between representatives of different species. The intra- and interspecies transfer was demonstrated for the SaPI-2 element which could be transferred into a variety of different recipients [22,25,26]. The identification of self-replicating plasmid-like states of the excised SaPI element, however, is also reminiscent of plasmid-like ancestors [22]. Bacteriophage-mediated transfer is limited by the amount of DNA that can be packed into the phage capsid, but in some cases it can expand beyond 100 kb [27,28]. As multiple island-like genomic regions in other bacteria exhibit features of degenerate prophages as well, there may be the possibility to mobilize these islands by other phages.

The discovery of integrative conjugative elements (ICEs) and related genetic entities suggests another mechanism of PAI transfer [29-32]. With the help of excisionases and integrases PAIs and related integrative mobilisable elements are able to site-specifically delete from or integrate into the chromosome. After deletion they are able to replicate and can also be transmitted into a new host by their own conjugative machinery. A variant of the "high pathogenicity island" (HPI) has been described in *E. coli *strain ECOR31 to contain a 35-kb sequence with striking homology to conjugative plasmids [33]. The identification of this ICE-EC1 carrying a functional transfer determinant suggests that conjugative transfer may have played a role in the spread of the HPI, and possibly also in the transmission of other PAIs. The spread of the non-selftransmissible but mobilisable antibiotic resistance gene cluster of the *Salmonella *genomic island 1 (SGI1) also supports the existence of a conjugal transfer mechanism for PAIs as well as interstrain PAI transfer observed in *Pseudomonas aeruginosa, Enterococcus faecalis *and *Streptococcus thermophilus *[34-36]. Type IV secretion systems (T4SSs) have been shown to mediate the horizontal transfer of such DNA elements in a broad range of bacteria [32,37-40]. Alternatively, (co-)mobilisation of circular intermediates of islands and related genetic elements has been described [23,41-44].

To study whether archetypal PAIs of *E. coli *which usually lack traits that enable their distribution such as origins of replication and *tra *genes could be generally (co-)mobilised by a helper plasmid, we investigated the transferability of PAI II_536_, the largest PAI (102.2 kb) of UPEC strain 536, into an *E. coli *K-12 recipient and back into a PAI II_536_-negative mutant of strain 536.

## Results

### Transfer of the entire PAI II_536 _from UPEC strain 536 into *E. coli *K-12

**Figure 1:**
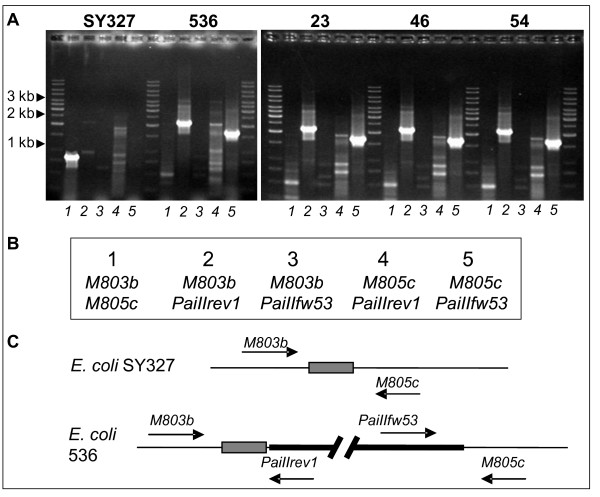
**Confirmation of the chromosomal insertion of the mobilised PAI II**_**536 **_**in recipient strain SY327**. *leuX *and PAI II_536_- specific PCRs were carried out (**A**) with laboratory K-12 strain SY327*λpir*, wild type strain 536, and the transconjugant clones 23, 46, 54. For this purpose, four test primers (*M803b, M805c, PaiIIrev1, PaiIIfw53*) were used in different combinations indicated in (**B**). The orientation of the primers relative to *leuX *(grey box) in a K-12 strain and in the wild type strain 536 is depicted in the lower part of the figure (**C**).

Altogether, 31 mating experiments were carried out at 20°C and 37°C. Plating of conjugation batches with *E. coli *strains 536-19/1mob (donor) and SY327λ*pir *(recipient) resulted in high numbers of chloramphenicol (Cm) and nalidixic acid (Nal)-resistant colonies and 899 resulting haemolytic clones were further investigated. Screening of clones that grew on the Cm-Nal selective blood agar medium with the *E. coli *K-12- and K15 capsule-specific PCRs, however, revealed that only 27.6% (248 clones) of them were true *E. coli *K-12 transconjugants, whereas the rest proved to be spontaneous nalidixic acid resistant mutants of strain 536. These clones were further analysed with four PAI II_536_-specific PCRs (Figure 1B) to determine whether the complete PAI II_536 _had been transferred. 93.1% (231 clones) of the 248 transconjugants acquired the complete island and 6.9% (17 clones) of the haemolytic transconjugant clones have only been partially transferred to the recipient strain.

The mating temperature slightly affected the proportions of the different types of PAI transfer. At 20°C, 81.5% (n = 88) of the transconjugants carried the chromosomally inserted PAI II_536 _construct, 14.8% (n = 16) had circular intermediates, and 3.7% (n = 4) resulted from partial PAI II_536 _transfer. Upon mating at 37°C, 70.0% (n = 98) of PAI II_536 _were chromosomally inserted, 20.7% (n = 29) were circular intermediates, and 9.3% (n = 13) were only partially transferred. The differences observed between the different types of transconjugants obtained at 20°C and 37°C were not significant.

Transfer frequencies were between 1 × 10^-7 ^and 6.66 × 10^-9 ^(data not shown), depending on the mating temperature (20°C or 37°C) as well as on the ratio of donor and recipient cells (3:1 or 9:1). The mean transfer frequency at both temperatures was always higher with a donor: recipient ratio of 9:1 relative to a 3:1 ratio. The differences observed were, however, not significant (Table 1).

**Table 1:** Mobilisation and remobilisation of PAI II_536_

	**Transfer rate of PAI II**_**536**_	
	20°C	37°C
Mobilisation rate from* E. coli *536 to *E. coli *SY327		
Donor-recipient ratio 3:1	3.47 × 10^-08 ^± 4.85 × 10^-09^	3.65 × 10^-08 ^± 5.46 × 10^-09^
Donor-recipient ratio 9:1	4.93 × 10^-08 ^± 1.14 × 10^-08^	4.31 × 10^-08 ^± 6.11 × 10^-09^
		
Remobilisation rate from *E. coli *SY327 to *E. coli *536-21		
Donor with integrated PAI II_536_	1.41 × 10^-07 ^± 1.25 × 10^-07^	8.00 × 10^-08 ^± 7.47 × 10^-08^
Donor with CI of PAI II_536_	4.32 × 10^-05 ^± 3.65 × 10^-05^	3.75 × 10^-05 ^± 3.18 × 10^-05^

31 and 10 independent conjugation experiments were performed for the mobilisation and remobilisation experiment, respectively. Plasmid RP4 was used as a helper plasmid for mobilisation of the excised PAI II_536 _construct from *E. coli *536 into recipient *E. coli *SY327.

### PAI II_536 _integrates site-specifically into the *E. coli *K-12 chromosome at the tRNA gene *leuX*

Upon conjugation, the transferred circularised form of the PAI II_536 _derivative can integrate into the recipient's chromosome. Additionally, the recipient strain SY327λ*pir *also enables episomal replication of the transferred CI. Analysis by PCR of the transconjugants carrying the complete PAI II_536 _derivative allowed to distinguish between chromosomally inserted and episomal circular forms of the PAI II_536 _construct. Episomal CIs could not be detected in the clones with the chromosomally inserted PAI II_536 _derivative. As exemplarily shown for clones 23, 46, and 54, the orientation of the site-specifically integrated PAI II_536 _within the chromosome was determined by using combinations of the four primer pairs indicated in Figure 1. In these three clones as well as in donor strain 536, PCR screening products could only be obtained using primer pairs 2 and 5, which amplify the ends of PAI II_536 _with the adjacent core genome context. Primer pair 1 amplifies the empty *leuX *locus in the core genome context and gave only a PCR product in the recipient strain SY327. Accordingly, PAI II_536 _has been inserted into the *leuX *gene of the *E. coli *SY327 chromosome in the identical orientation as in the donor chromosome (Figure 1).

**Figure 2:**
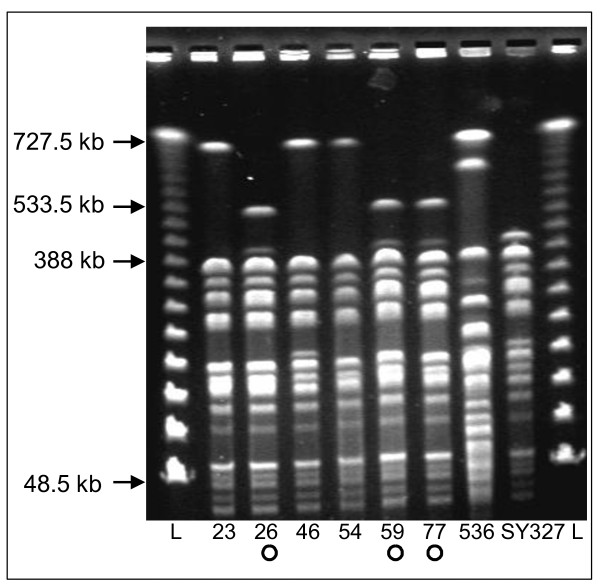
**Analysis of the genomic restriction pattern of different recipient clones upon transfer of PAI II**_**536 **_**by PFGE**. Genomic DNA of three representatives of the transconjugants carried either a chromosomally inserted PAI II_536 _or an episomal circular intermediate (CI) were digested with *Sfi*I. Donor strain 536 and recipient strain SY327*λpir *are controls. Recipients 26, 59, and 77 (marked with '**o**') carried a PAI II_536_-specific CI, whereas in strains 23, 46, and 54 PAI II_536 _has been chromosomally inserted at the *leuX *tRNA locus. L, Lambda Ladder PFGE marker, (New England Biolabs).

Genomic restriction patterns of representative transconjugants, carrying either the chromosomally inserted PAI II_536 _derivative or its episomal CI, were compared to each other and to those of the donor and recipient strain by PFGE in order to assess their genomic homogeneity (Figure 2). Generally, the restriction patterns of the transconjugants were very similar to that of recipient strain SY327λ*pir*. The PFGE patterns of the selected transconjugants which carried the transferred PAI II_536 _in their chromosome exhibited only minor differences among each other. Similarly, the restriction patterns of the clones containing the stable episomal CI of PAI II_536 _were identical. Both groups of transconjugants could be clearly distinguished upon the presence of a ~400-kb and a ~530-kb restriction fragment in those recipient clones with a stable cytoplasmic PAI II_536 _CI which were absent from recipients in which chromosomal integration of the island occurred. Instead, a restriction fragment of about 700 kb was visible in the latter clones (Figure 2). This larger restriction fragment may comprise the 530-kb restriction fragment after chromosomal insertion of the transferred PAI II_536 _(107-kb) construct. These data demonstrate that PAI II_536 _can be mobilized upon excision from the chromosome by helper plasmids into suitable recipient strains. Upon transfer, the majority of CIs integrates site-specifically into the recipient's chromosome at the *leuX *locus or remains as an episomal CI.

### Remobilisation of the transferred PAI II_536 _into *E. coli *strain 536-21

Since two types of transconjugants resulted from the PAI II_536 _mobilisation, two types of remobilisation experiments were performed: K-12 strains harbouring either the CI or the chromosomally inserted PAI II_536 _were used as donors. Since the recipient strain 536-21 does not express the π-protein, only chromosomal integration of PAI II_536 _into the *leuX *gene was observed in all transconjugants. There was a marked difference in the conjugation efficiency between the remobilisation of the circular and the integrated forms. In those cases where strain SY327-77 carrying an episomal CI of PAI II_536 _was used as donor, average PAI transfer was about 100- to 1000-fold more efficient with transfer rates of 3.75 × 10^-5 ^at 37°C and 4.32 × 10^-5 ^at 20°C, respectively. However, if SY327-54 served as a donor, where PAI II_536 _was integrated into the chromosome, the average efficiency of transfer was 8 × 10^-8 ^and 1.4 × 10^-7^, at 37°C and 20°C, respectively (Table 1). These results support that the mobilised PAI and the RP4 plasmid include all the factors required for excision of the chromsomally inserted PAI as well as for its efficient transfer.

## Discussion

Horizontal gene transfer (HGT) plays an important role during prokaryotic evolution. Exchange and accumulation of a variety of fitness or virulence factors frequently carried on mobile genetic elements contributes to evolution of different pathogens and pathotypes from non- or less pathogenic variants [8,45]. One perfect environment for this evolutionary process is the mammalian gut with its large bacterial density which offers the possibility of close cell-to-cell contacts between closely or even remotely related bacteria. In this way, members of the gut flora, such as *E. coli*, may also increase their pathogenic potential and may evolve from commensals into e.g. extraintestinal pathogens. *E. coli *may, nevertheless, also exist outside of the gut, e.g. in the environment having the possibility to exchange genetic information with other bacteria. High bacterial cell densities could be observed, e.g. in bacterial biofilms, an important bacterial lifestyle in the environment. The PAI II_536 _transfer at 20°C indicates that *E. coli *can exchange PAIs not only upon growth at human body temperature but also at a temperature which is closer to the ambient temperature in the environment.

For the transfer of PAIs, different mechanisms have been postulated. For example, the presence of phage-related sequences on most PAIs suggests a key role of bacteriophages in HGT, and, indeed, a transfer by transducing phages has been reported for VPI and SaPI1 [20,26,27]. Flanking direct repeat sequences (DRs) and an active bacteriophage integrase play also an important role in the excision process of *E. coli *536-specific PAIs [18], which is essential for a subsequent transfer. Alternatively, PAIs can be transfered by conjugation. The HPI of *E. coli *strain ECOR31 with its flanking DRs, an integrase gene and the right border region (RB-HPI_ECOR31_) encoding a functional mating pair formation system and a DNA-processing region, fulfills all structural criteria of integrative and conjugative elements, ICE [29,31,33]. Although neither conserved *repABC *genes, other indications of a plasmid replicon, nor mobilisation have been detected, this HPI variant supports the hypothesis that PAI transfer can also occur by conjugal transfer [33]. Furthermore, high partial similarity between different polyketide biosynthesis determinants located on islands such as the HPI and the colibactin island of extraintestinal pathogenic *E. coli*, ICEs and different enterobacterial plasmids have been previously described. The presence of these polyketide determinants in different enterobacterial species and their (co-)localisation on different mobile genetic elements further support the idea that different chromosomal and episomal elements can recombine and thus due to HGT promote bacterial genome plasticity [46]. Additionally, self-transmissible conjugative elements can mobilize other genomic DNA regions in *cis *or in *trans*. The conjugative plasmid RP4, for example, can mediate transfer of mobilizable plasmids which code for an origin of transfer (*oriT*), a relaxase and nicking accessory proteins for interaction with *oriT*. A conjugative element then provides the mating pair formation functions for transfer [47].

Large-scale DNA transfer followed by homologous recombination can also be involved in the distribution of chromosomally inserted pathogenicity islands. Different HPI-transfer events have been detected in *E. coli*, in which not only the HPI itself but also flanking regions of the genomic backbone have been transfered. Schubert and colleagues demonstrated that the conjugative F plasmid can transfer and insert the HPI into the recipient chromosome by homologous recombination of flanking DNA regions. Upon chromosomal integration of an F plasmid, the recipient genome acquires an *oriT *and thereby becomes mobilisable. Resulting so-called "high frequency of recombination" (Hfr) strains can transfer large parts of their chromosomes at high frequency [13].

PAI deletion has been described for UPEC strain 536 and other pathogenic bacteria [10,14,17,48-50] as well as the occurrence of circular intermediates upon PAI excision of [12,23,26,30,33,35,36,50] suggesting that the latter could be formed during conjugal or phage-mediated transfer. Using a conjugative helper plasmid, transfer of a CI was also verified for the 43-kb *Salmonella *genomic island 1 (SGI1) [30]. In addition, the 35-kb HPI of *Yersinia enterocolitica *could be mobilised [51] when a modified RP4 plasmid was used as a shuttle vector during the transfer experiments. Several cases of plasmid mobilisation as a major mechanism for horizontal gene transfer of PAIs have been described [42-44].

With the PAI II_536 _construct used in this study, we were able to transfer this ~107-kb DNA region in the presence of the unmodified RP4 plasmid and thereby demonstrated that PAI II_536 _is mobilisable, but not self-transmissible. To increase the stability of the large PAI II_536_-specific CI and thus the transfer frequency, we also integrated an origin of replication into this PAI. In this respect, our model construct is artificial, but exhibits similar features of some ICEs including the HPI_ECOR31_. In the latter case, the origin of replication seems to be inactivated by insertion of an IS*630 *homologue [33]. This may explain why HPI_ECOR31 _is not transferable although CI formation of this island was shown in the same study. Whereas plasmids replicate autonomously, ICEs are generally thought to be incapable of autonomous replication. Instead, their replication depends on that of host chromosome [52]. Some ICE and ICE-like elements, however, have been reported to be capable of autonomous replication [53-57]. In the light F plasmid-mediated mobilization of the HPI [13], it would, nevertheless, also be interesting to analyse in the future if a PAI II_536 _construct, which is not a self-replicating entity, but only carries an *oriT*, could be mobilized upon provision of the appropriate conjugative machinery *in trans *on a plasmid.

The primary aim of our study was to demonstrate the transferability of a large archetypal island of UPEC strain 536 as this PAI can be excised site-specifically from the chromosome by its cognate integrase. On the other hand, we also tested conditions which may affect the transfer of an excised circular PAI intermediate. The frequency of PAI transfer in the mobilisation experiments was low (between 10^-8 ^and 10^-9^). We postulate that the efficiency of PAI II_536 _transfer depends on several factors including the growth temperature, integrase activity, the size, and the chromosomal or episomal state of the PAI. In spite of the large size of PAI II_536_, complete transfer occurred at a high rate. 93.1% of the transconjugants received the complete 107-kb PAI II_536 _construct. The activity of the PAI-encoded integrase can contribute to the transfer efficiency by affecting the PAI excision as well as the integration frequency. The remobilisation efficiency was three log scales higher with a stable episomal CI compared to an integrated PAI, indicating that a more active integrase may increase the chance of transfer by frequent induction of PAI-excision from the chromosome (Table 1). PAI II_536 _transfer rates at 20°C and 37°C were not significantly different. Besides the gut, *E. coli *also faces the environment as a natural habitat since the bacteria are excreted each day in considerable amounts. As a part of naturally occurring biofilms in sewage or drinking water systems, they are exposed to stimuli described above, i.e. low temperature and high density of cells, what might explain their ability to efficiently exchange genetic elements also under these conditions.

In accordance with previously published results [18], the mobilisation and remobilisation experiments corroborated that the P4-like integrase of PAI II_536 _is highly specific. In both strain backgrounds, SY327*λpir *and 536-21, the PAI II_536 _was found only to be inserted into the *leuX *locus thereby restoring the complete tRNA gene in the latter strain. This result demonstrated that *leuX *is the preferred chromosomal integration site of PAI II_536_. Site-specific chromosomal integration of PAIs has already been described before. However, if multiple isoacceptor tRNA genes exist, chromosomal insertion may occur at all the available isoacceptor tRNA loci. The HPI of *Y. pestis *is usually associated with the *asnT *tRNA locus, but in *Y. pseudotuberculosis *the HPI can insert into any of the three chromosomal *asn *tRNA loci [58]. The same phenomenon has been observed as well, e.g. with LEE PAIs [12] and the PAPI-1 island of *P. aeruginosa *[36].

The lack of genes required for mobilisation and/or transfer on the archetypal PAIs of UPEC strains such as *E. coli *536 has been considered to reflect an advanced stage of "homing" of these islands, i.e. an ongoing process of stabilisation of such chromosomal regions resulting from the selective inactivation and loss of corresponding genes [5,32]. Consequently, horizontal transfer of such islands, although they can be efficiently excised from the chromosome, could not be detected so far and the mechanism of acquisition remains speculative. This study further supports the important role of mobilisation and conjugation for transfer and dissemination of genomic islands and indicates that loss of mobilisation and transfer genes promotes stabilisation of horizontally acquired genetic elements in the recipient genome.

## Conclusions

We provide evidence that a 107-kb chromosomal PAI derivative of UPEC can be mobilised into other *E. coli *recipient strains. This transfer was dependent on the presence of a helper plasmid and accessory transfer genes. The new host with the mobilisable PAI II_536 _could also serve as donor passing on this PAI to other recipients. These results underline that in a suitable genetic background dissemination of large genomic regions such as PAIs by conjugal transfer contributes to genome plasticity of *E. coli *and the evolution of bacterial pathogens. Stabilisation of beneficial genetic information localised on mobile genetic elements can be achieved by selective loss of transfer or mobilisation functions encoded by these elements.

## Methods

### Bacterial strains and growth conditions

The complete list of the strains and plasmids used in this study is shown in Table 2. Analysis of the complete genome sequence of *E. coli *strain 536 (O6:K15:H31) revealed the presence of six large typical PAIs [59]. For the mobilisation experiments strain 536-19/1mob was used as donor, and the laboratory strain SY327λ*pir *[60] served as recipient. Two different donor strains were used for re-mobilisation of PAI II_536_. In strain SY327-77, the mobilised PAI II_536 _existed extrachromosomally as a circular intermediate. In strain SY327-23, the transferred island was chromosomally inserted at the *leuX *tRNA gene. Strain 536-21, a PAI I_536_- and PAI II_536 _deletion mutant was used as recipient for remobilisation.

**Table 2:** Bacterial strains and plasmids

Strain or plasmid	Relevant characteristic(s)	Source or reference
***E. coli *strains**		
536 wt	UPEC wild type strain, Sm^R^	[69]
536-21	536, ΔPAI I_536 _ΔPAI II_536_, Sm^R^	[2]
536-19/1mob	Donor strain in the mobilisation experiments, *pir*_*λatt*_*, mob*_GP704 _inserted in PAI II_536_, pRP4, Sm^R^, Ap^R^, Cm^R^, Tc^R^, Km^R^	This study
SY327λ*pir*	F^-^, *araD*, Δ(*lac pro*), *argE*(Am), *recA56*, Rif^R^, *gyrA λpir*	[60]
SM10λ*pir*	*thi*1, *thr*1, *leu*B6, *sup*E44, *ton*A21, *lac*Y1, *recA*::RP4-2-Tc::Mu λ*pir *Km^R^	[60]
SY327-23	Mobilised PAI II_536 _is integrated into *leuX*	This study
SY327-77	Mobilised PAI II_536 _is present as a CI	This study
**Plasmids**		
pGEM^®^T-Easy	*bla*, T/A cloning vector	Promega
pGP704	*bla, ori*R6K, *mob*RP4	[60]
pSG704	*cat, ori*R6K, *mob*RP4	This study
pCVD442Tc	*bla, tet, ori*R6K, *mob*RP4, *sacB*	This study
pLDR8 *neo, int *expression vector, Ts	*neo, int *expression vector, Ts	[62]
pLDR9	*bla neo*, cloning vector to integrate DNA into *attB*	[62]
pPAI II-CI	*bla*, positive control for detection of PAI II_536_-specific CIs	[17]

Bacteria were grown in Luria broth (LB) or on LB agar at 20°C or 37°C. In the mobilisation experiments, selection of transconjugants was performed on blood agar plates, whereas lactose containing M9 minimal agar plates were used for the remobilisation experiments. If required, antibiotics were used in the following concentrations: ampicillin (100 μg/ml), chloramphenicol (20 μg/ml), kanamycin (100 μg/ml), streptomycin (10 μg/ml), tetracycline (5 μg/ml) and nalidixic acid (4 μg/ml).

### Oligonucleotides

The list of oligonucleotides used in this study is compiled in Table 3. Oligonucleotides were purchased from MWG Biotech (Ebersberg, Germany) or Sigma-ARK (Steinheim, Germany).

**Table 3:** Primers used in this study

Designation	Sequence (5` - 3`)	Comment and reference
	Primers for the mobilisation experiments	
Cm_fw_PstI	CTGCAGGCTTGTCAGGGGGGCGGAG	*cat *cassette in pGP704
Cm_rev_PstI	CTGCAGACCCTGCCCTGAACCGACGA	"
pir_fw_SacI	TTTGAGCTCCCGTCAAGTCAGCGTAATGCTC	Introduction of a stop codon into *pir*
pir_revStop_ EcoRI	TTTGAATTC*TCA*AGCTTCATCATCTTTATCGCCAGA	"
paiII_1XhoI	TTTCTCGAGGGGAAGCACGATATGCAGCC	Labelling of PAI II_536 _by homologous recombination with pSG704
paiII_1SacI	TTGAGCTCGATATTTTTTGGCTGCTTCAGCTTTACG	"
paiII_2SacI	TTTGAGCTCGGACAAGAACACAAAATCACTCTACTGA	"
paiII_2XhoI	TTTCTCGAGCCCGGCTTCATCGACAATGA	"
ATT1	GAGGTACCAGCGCGGTTTGATC	Confirmation of *pir *integration into the λ attachment site (*E. coli*)
ATT2	CAGATGGCGCAATGCCATCTGG	"
17 kD up	CCCGGCTGAACAGACGATT	Screening PCRs for PAI II_536_
17 kD in	GCAGCGGAGAGTCATTGTC	"
hlyDup	CGCGATAATCCGCTACATC	"
hlyDin	GGGTATGGCTGTCACTGCA	"
hec_down1	CACACTGAAAGGCCGCAGC	"
hec_down2	GTACAGCGTGCCGTTCTGC	"
dsdXin	GCTGTATCCCGACATCAGC	"
dsdAup	GCCACATCATTCTCCCGTA	"
ORFAin	CATCCCCTTCACTACAGGA	"
Na-Anti_pdo	CAGCGATGCTGGACCGTAT	"
PaiII_1fw	CAACCGTAGAAATACGTGCCG	Overlapping PCRs of PAI II_536_
PaiII_1rev	CCAGTATGAGGCAAACCCTAAAG	"
PaiII_2fw	GACAATAGCTGTCCATACGGTG	"
PaiII_2rev	CAATGCTGGCCATATCCATCAG	"
PaiII_3fw	GACTCACTACTGGGAAGGTC	"
PaiII_3rev	GATGGTCATGTGCAGGGAGG	"
PaiII_4fw	GTGATGTCACTGGCGGTAATAATC	"
PaiII_4rev	GGCGATGACCATGATACGGTA	"
PaiII_5fw	CGGAATACTGAACTGCGGATAA	"
PaiII_5rev	GCTGGATGCCAATCAATGATCG	"
PaiII_6fw	GCATCCATCTCCGTTCACAG	"
PaiII_6rev	GCATGGCTGGTTGTTCCTAAAC	"
PaiII_7fw	CCTCCTTTGGACTGAAGTTCA	"
PaiII_7rev	GCACAGGCGCTCTTTTATTGTTTG	"
PaiII_8fw	GGACACATGGCAGGGTCTG	"
PaiII_8rev	GGAACACGTCTTCTTGTTGACA	"
PaiII_9fw	CAGCGATTTGTCACCACCTG	"
PaiII_9rev	GTACTTGACGCGGCGGACA	"
PaiII_10fw	GCTTCTGAAAAACGGGTGAAGTC	"
PaiII_10rev	CATGGCGCATCATGAAATCATCA	"
PaiII_11fw	CTCTCGCGTATATTCAGCAAAAAC	"
PaiII_11rev	CGTCACATCGGATAACATTCGG	"
PaiII_12fw	CTGATATCTCTTTCAGACTTCAGAAC	"
PaiII_12rev	GTCACCTCAAGAACGTCTAACC	"
PaiII_13fw	CTGGCACCTATGGATCAGGT	"
PaiII_13rev	GTTCAGCAACTGAAGGTTCATACT	"
PaiII_14fw	GATGACCATCAGTGTTTCCGCT	"
PaiII_14rev	CGGGGATTTAAGTATTGGTCAGTT	"
PaiII_15fw	GCAACAATCTGACCTGCAAGCAT	"
PaiII_15rev	GGATGATGAGCTTCAGGTTCAG	"
PaiII_16fw	TGCCGTACAGCTTGTCATTACC	"
PaiII_16rev	CTGGGTACTGCACTTTCCTCA	"
PaiII_17fw	CTGACATTGCCACCAGATTTTTGT	"
PaiII_17rev	GGTGTAATGCGCTAACCTGTTTC	"
PaiII_18fw	CTACAAATGTTCAATATGGTGGGTATATC	"
PaiII_18rev	CGCTGTTGCCACTGGATTAATG	"
PaiII_19fw	GCCATCCACTACATATCATGCC	"
PaiII_19rev	CGACGGGTTTCTATGCTGAG	"
PaiII_20fw	CCTCAACTGGAGCAATTTTCTGTC	"
PaiII_20rev	GGACTTGGATCACTGAAGCTTTAC	"
PaiII_21fw	CCACAAGCTGTTGATTTTGGTACG	"
PaiII_21rev	GCGATAGTGGGCAATTTGCTATTG	"
PaiII_22fw	CGTAAACGTCCTCCAGAATTTATATC	"
PaiII_22rev	GGACGATGGCGATATGTCTG	"
PaiII_23fw	GTTCTTCATTTCTACGTTGCTTTGTC	"
PaiII_23rev	CCTACCAGAGATAACCCATGG	"
PaiII_24fw	CGGTTTCTCCTGAACATAACTTTG	"
PaiII_24rev	GGTGAAGTCCGTAACCAGAATG	"
PaiII_25fw	GCCTGTTTTTGCTGCTGTTCAC	"
PaiII_25rev	GCAGGACTAAAGTTGCAGAGC	"
PaiII_26fw	CGCTTTCGCCCCGATTTCTA	"
PaiII_26rev	ATGACCGTCGTACTGTGGAC	"
PaiII_27fw	GTCAGCCCGCTTTTCTTCTG	"
PaiII_27rev	GCTCCGTCGTATACCGATGA	"
PaiII_28fw	CGGTCAAGAAAATACGATGAGCC	"
PaiII_28rev	GAACGACAGCAAAATCCTCTCC	"
PaiII_29fw	CAGCACCTGCGCCGTCA	"
PaiII_29rev	GCGATGCCACGGTGAAAACC	"
PaiII_30fw	CCGGTATTACTGAATGTCCCG	"
PaiII_30rev	GAACATGAAGACAGCACTGACC	"
PaiII_31fw	CCTGAAGCAGAACATCATCCAG	"
PaiII_31rev	CGGCTGATATCCTGAGACTG	"
PaiII_32fw	CTCGCTTCCACGGACGTTG	"
PaiII_32rev	GTTGGCAGTGCTGAAAGCAG	"
PaiII_33fw	GGTCAGAATGTCCTCAGTGAG	"
PaiII_33rev	CAATGAAATTGACAGGAGAGATACC	"
PaiII_34fw	CCACTGGCATGATTTTTACCCTG	"
PaiII_34rev	CGGGCACATTCCTGCTGTC	"
PaiII_35fw	GGATACTGGGCGATGAGCG	"
PaiII_35rev	GAGCACGGTGAGCGAAATAG	"
PaiII_36fw	CCGCATTAGGTGACTTTACACG	"
PaiII_36rev	GACGCCGTACTGACCGATG	"
PaiII_37fw	CCAGGTTTGTTATCGAGGTAAGG	"
PaiII_37rev	GGCGCTATCGACTACGTCC	"
PaiII_38fw	GGCAGTATATCGATTCGGCGA	"
PaiII_38rev	GCTTCCCAGCCTGTCACTTC	"
PaiII_39fw	GGGCATCTTCAAAGTCAAAGCC	"
PaiII_39rev	CGCCCGTCTGTTTTTCAGTTTC	"
PaiII_40fw	GGGGCATCAAGGTCGCTATTT	"
PaiII_40rev	CAGAACCGCAGCCAGCCAT	"
PaiII_41fw	GCTGCGATGCGGATCCAC	"
PaiII_41rev	GGTTACCGCAATGGTGAAAGG	"
PaiII_42fw	GCTTTTACTGCGCCGACATCA	"
PaiII_42rev	CGTTGCACGCGGCTATCTG	"
PaiII_43fw	CGATGGATACATTCGGGTTTAGC	"
PaiII_43rev	GCAACAGCGACATCATCCTG	"
PaiII_44fw	CTCTCTCTTCAGCCAGTCATC	"
PaiII_44rev	GCCAAAATCTGATCCCCAGC	"
PaiII_45fw	GCAACTACGCCATTGGTTTGTC	"
PaiII_45rev	GAAAAACTGGCACGTCATCAACG	"
PaiII_46fw	GAAGGCTGCCATTCGGGTATA	"
PaiII_46rev	CTGTACTGACTCGTCAGCACT	"
PaiII_47fw	CTTGAGATTCAGCAAGGTGGC	"
PaiII_47rev	GGAATCCCCTAATGCTGGTG	"
PaiII_48fw	GTATAACGGGATGAAAGTGGGG	"
PaiII_48rev	GTTGAGAATGTCGGGAATGGTAC	"
PaiII_49fw	GGATGTGTATCAGACAAAGCAATG	"
PaiII_49rev	TTTCTGGCGAATTTCTTCAGGAAG	"
PaiII_50fw	CAGCCATTTTTCCCTCTCCG	"
PaiII_50rev	CCTGACCATCTTCCGTCATG	"
PaiII_51fw	CTGCTGTTCACTGTGGCATC	"
PaiII_51rev	GAGTGGCAACCAGTTGAGACT	"
PaiII_52fw	CGCATAATTCCACCACACCTTC	"
PaiII_52rev	GGCTGGTCGGTACGCAC	"
PaiII_53fw	GGCAGGCATTTCACTGTGTGA	"
PaiII_53rev	CGAAGGCCGGACTCGAACA	"
K12R (rev)	ATCCTGCGCACCAATCAACAA	*E. coli *K-12 specific [67]
K12L (fw)	TTCCCACGGACATGAAGACTACA	"
K12IS-L (fw)	CGCGATGGAAGATGCTCTGTA	"
orf4bico	GGAATGAATGCCACTCCATTATTGACAGAAATG	*E. coli *536 (K15 capsule)- specific
orf5bico	GATCAAACGAGTCAGCTAAATAATCCCCAC	*E. coli *536 (K15 capsule)- specific
M803b	GCCTGGAGTGTGACAAAGGTTAC	*leuX *flanking primers [17]
M805c	GATGTTCACCAAGGTGGGCGT	"
leu 2	ACCAAGCGCTGCAAAAAGAT	Concatemer PAI II_536_
Concat 1	CCGGATTGGATCTATCGCGA	"
yjgB1	ACTTTATCGGCACCCATCG	Downstream of *leuX*
yjgB2	GCATGAGGTGATTGGGCG	"

### Preparation and manipulation of DNA

Plasmid DNA and chromosomal DNA were isolated according to standard protocols [61]. Recombinant DNA manipulations were carried out with enzymes supplied by GE Healthcare (Freiburg, Germany) or New England Biolabs (Frankfurt am Main, Germany) according to the manufacturer's instructions and standard procedures [61].

For cloning experiments, the Expand Long Template PCR System including a DNA polymerase with proof reading activity was used (Roche Diagnostics, Mannheim, Germany) and PCR screenings were performed with the REDTaq^® ^ReadyMix™ (Sigma, Deisenhofen). PCR products were subcloned into the pGEM^®^T-Easy cloning vector (Promega, Mannheim, Germany).

### Construction of a mobilisable PAI II_536 _construct

**Figure 3:**
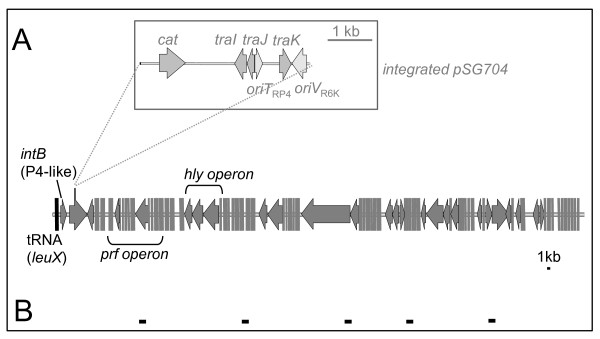
**Genetic structure of PAI II**_**536**_. For the transfer experiments, suicide vector pSG704 which carries the chloramphenicol acetyltransferase (*cat*) gene, an origin of replication and mobility genes (depicted in the enlarged insert) was stably integrated into a non-coding region of this island (A). Complete transfer of PAI II_536 _into the transconjugants was confirmed by detection of five regions of PAI II_536 _by PCR (B).

**Figure 4:**
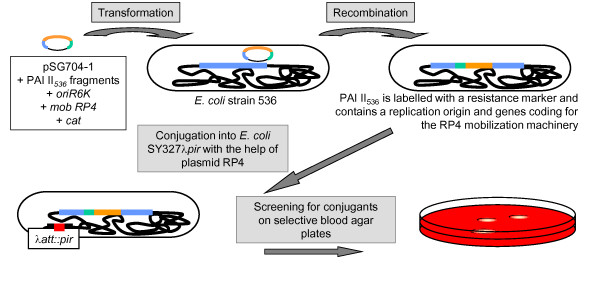
**Schematic presentation of the main steps of the PAI II**_**536 **_**mobilisation experiment**.

Introduction of a chloramphenicol resistance marker, mobilisation genes, an origin of replication, and an origin of transfer into PAI II_536 _was accomplished with the help of plasmid pSG704 which is a chloramphenicol-resistant derivative of the conjugative suicide vector pGP704 [60]. Prior to the insertion of the chloramphenicol acetyltransferase (*cat*) cassette into pGP704, the vector was digested with *Pst*I. Thereby, a 700-bp fragment that encompasses two thirds of the ampicillin resistance gene *bla *was deleted and replaced by the *cat *cassette that was amplified from pACYC184 with flanking *Pst*I sites. pSG704 resulted from ligation of two PCR products that correspond to non-coding sequences of PAI II_536 _located 2,500 bp downstream of *leuX *(amplified with the primer pairs paiII_1XhoI/paiII_1Sac and paiII_2Sac/paiII_2XhoI) into a *Sac*I restriction site of this plasmid. Homologous recombination between these 4.4-kb pSG704-derived DNA and PAI II_536 _resulted stable integration of the *cat *cassette, the *mob*_RP4 _region with the *traIJH *genes, the *oriT*_RP4_, and the *oriV*_R6K _in PAI II_536 _(Figures 1A, 3, 4). This replication origin is only functional in the presence of the bacteriophage lambda π-protein.

### Integration of the *pir *gene into the λ attachment site of uropathogenic *E. coli *strain 536

To stabilise the circular intermediate of PAI II_536 _after excision from the chromosome and thus enhance its transfer efficiency, we integrated the *pir *gene coding for the replication factor (π-protein) of the pSG704 *oriV *into the chromosomal λ attachment site of *E. coli *strain 536 (Figure 4). For this purpose, the *pir *gene was amplified from *E. coli *strain Sm10λ*pir *with the primers pir_fw_SacI and pir_revStop_EcoRI. A resulting 950-bp PCR product comprising a truncated, but functional π-protein was subcloned into pLDR9 [62] using *Eco*RI and *Sac*I. The resulting plasmid was used for *pir *integration into the λ attachment site as described before [62]. The correct *pir *integration was confirmed by PCR (primers ATT1 and ATT2). Expression of the active π-protein was confirmed by episomal propagation of a tetracycline-resistant derivative of the π-dependent suicide plasmid pCVD442 [63] in such strains.

### Mobilisation of the labelled PAI II_536 _by the broad host range conjugative plasmid RP4

Plasmid RP4 was shown to be able to efficiently mobilise the IncQ plasmid RSF1010 which only encodes relaxosomal components [64]. After introduction of the *mob*_RP4 _region coding for the TraI, TraJ and TraK proteins, which form the relaxosome at *oriT*, and the *oriV*_R6K _into PAI II_536_, the RP4 plasmid was conjugated into the corresponding recombinant strain (Figure 4) since the mating pair formation (Mpf) system of a conjugative plasmid is also necessary for a successful PAI or CI transfer [65,66]. The resulting strain was designated *E. coli *536-19/1mob. For further experiments, this clone was chosen as donor strain of the tagged PAI II_536_. The influence of the RP4 plasmid on PAI II_536 _instability was determined under different growth conditions. The deletion frequency of the island was not affected by the presence of RP4.

### Conjugative transfer of PAI II_536_

Conjugation was carried out on LB agar plates under non-selective conditions. Donor and recipient strains were grown separately until late logarithmic growth phase and were then mixed with each other according to the following procedure. Donor and recipient strains were adjusted to a ratio of 3:1 or 9:1, were centrifuged and resuspended in LB medium to a final volume of 0.1 ml. This mixture was spotted on a dry agar plate and incubated at 20°C and 37°C, respectively. These temperatures were chosen to represent the environmental growth temperature or the human body temperature. The plates were incubated for two days. During the mobilisation experiments (donor: 536, Sm^R^; recipient: SY327, Nal^R^), selection for transconjugants was performed on blood agar plates containing chloramphenicol (20 μg/ml) and nalidixic acid (100 μg/ml). In the remobilisation experiments (donor: PAI II_536 _containing derivatives of *E. coli *SY327, Nal^R^, Cm^R^; recipient: 536-21, Sm^R^) selection of clones with the remobilised PAI II_536 _was performed on M9 lactose medium containing streptomycin (10 μg/ml) and chloramphenicol (20 μg/ml). The frequency of transfer was calculated as follows: number of transconjugants/number of recipients.

### Analysis of candidate transconjugants for PAI II_536 _transfer, deletion, and integration

A thorough analysis of the transconjugants obtained was necessary, because spontaneous nalidixic acid-resistant mutants of strain 536 could occur. Clones that appeared on Cm-Nal blood agar plates were analysed by a four-step PCR process. In the first step, clones were tested with two *E. coli *K-12 specific primer combinations (K12R/K12L or K12R/K12ISL [67]) and with the strain 536-specific primer combination (orf4bico/orf5bico [68]). The latter primer combination amplifies a 1.5-kb fragment that is specific for the region 2 of the K15 capsule locus. Clones that were positive with the K-12-specific primers and negative with the K15 capsule gene-specific primers, i.e. putative *E. coli *K-12 recipients, were additionally tested with PAI II_536_-specific primers in the second step. To confirm the presence of the transferred PAI II_536_, five primer pairs (17 kDup/17 kDin, hlyDup/hlyDin, hec_down1/hec_down2, dsdXin/dsdAup, ORFAin/Na-Anti_pdo) were used which amplify 800 to 1600-bp fragments of different regions of the PAI II_536 _(Figure 1B). Those clones that were positive in all five screening PCRs were subjected to a more detailed PCR analysis to verify transfer of the entire PAI II_536 _and to exclude possible internal deletions of the transferred PAI II_536_. For this purpose, 51 PCRs were designed to amplify overlapping sections of PAI II_536 _with an average size of 2 kb, thereby covering the entire PAI II_536_. In addition, transconjugants were tested for CI formation (PaiII_1rev/PaiII_53fw) and for site-specific integration of PAI II_536 _into the tRNA gene *leuX *(M803b/M805c). The latter two primer pairs also allowed the determination of the orientation of the integrated PAI (Figure 2).

Remobilization experiments were carried out with two PAI II_536_-positive clones of *E. coli *K-12 as donors that derived from the mobilisation experiments and a derivative of the wild type UPEC strain 536 as recipient. Donor and recipient strains were mixed in a 3 : 1 ratio and incubated at 20°C and 37°C, respectively. Experiments were divided into two sets according to the state of the mobilised PAI. In the first set, the donor strain SY327-77 harbored PAI II_536 _in the circular form. In the second set, clone SY327-23, harboring the chromosomally integrated PAI II_536_, served as donor strain. In both cases, strain 536-21, a non-hemolytic derivative of strain 536, which lacks the two islands encoding functional α-hemolysin determinants (PAI I_536 _and PAI II_536_) [2], served as the recipient. In the remobilisation experiments, the same PCR-based verification process as described above was carried out with exconjugants that grew on the Cm-lactose-M9 minimal agar plates. In addition, pulsed-field gel electrophoresis (PFGE) analysis of the randomly picked transconjugants was carried out. Genomic DNA for PFGE analysis was prepared and cleaved with *Not*I or *Sfi*I as described before [2]. Gels were run for 21-24 h with pulse times of 0.5-50 s.

### Phenotypic characterisation of transconjugants

PAI II_536 _comprises a α-hemolysin gene cluster. This determinant was used as a phenotypic marker in this study to verify the presence of PAI II_536 _in transconjugants after the mobilisation and remobilisation experiments. Therefore, transconjugants were screened post-experimentally on blood agar plates to analyse the hemolytic activity. UPEC strain 536 served as a positive control, while strains SY327 and 536-21 served as negative controls.

### Statistical analysis

Statistical analysis of the conjugation rate was performed by the Mann-Whitney U test. The ratio/distribution of integrated, cointegrated and partial transconjugant clones at 20°C and 37°C was compared by the chi-square test. The difference was considered significant if p < 0.05.

## Authors' contributions

GS and UD designed research together with BH, LE and JH. GS constructed the mobilisable PAI II_536 _variant and performed the mobilisation and transconjugation experiments assisted by VS. BM and BH provided bacterial strains and constructs and supported the construction of the mobilisable PAI II_536 _variant, suitable recipient strains as well as mobilisation experiments. GS and UD wrote the manuscript assisted by BM, LE and JH. All authors have read and approved the final manuscript.
